# Racial and Gender Disparities for Glaucoma Treatment Rates in Upstate New York

**DOI:** 10.3390/jcm13237225

**Published:** 2024-11-28

**Authors:** Karen Allison, Brittany Hodges, Mohammed Mehdi Shahid, Changyong Feng

**Affiliations:** 1 Flaum Eye Institute, University of Rochester, Rochester, NY 14642, USA; brittany_hodges@urmc.rochester.edu (B.H.);; 2 Prevention of Blindness from Glaucoma and Age Related Macular Degeneration, 248 36 Jericho Turnpike, Floral Park, NY 10012, USA; 3 Department of Biostatistics and Computational Biology, School of Medicine and Dentistry, University of Rochester, Rochester, NY 14642, USA; changyong_feng@urmc.rochester.edu; 4 Department of Anesthesiology and Perioperative Medicine, University of Rochester, Rochester, NY 14642, USA

**Keywords:** glaucoma, glaucoma treatment, disparities in glaucoma care, blindness from glaucoma

## Abstract

**Introduction:** Glaucoma is one of the leading causes of irreversible blindness around the world. Black individuals are two times more likely to be diagnosed with glaucoma compared to White individuals. In 2019, the prevalence of glaucoma in Monroe County was highest amongst older individuals aged 85 and non-Hispanic Blacks. This study seeks to explore differences in glaucoma treatment rates that may be exacerbating disease severity and prognosis for individuals most acutely affected by glaucoma in Monroe County, NY. **Methods:** We used data from the Center for Disease Control’s National Vision and Eye Health Surveillance System (VEHSS) to assess the rates of glaucoma treatment for different racial, gender, and age demographic groups in Monroe County, NY. The source data were from individuals enrolled in Medicare who filed a claim. We stratified the data based on the glaucoma treatment type: laser glaucoma surgery, glaucoma drainage devices, other glaucoma surgery, or glaucoma prescription drugs. The main outcome variable was the prevalence rate of various types of glaucoma treatment in Monroe County, NY. The data were analyzed by potential risk covariates such as race/ethnicity, age, and gender. A descriptive data analysis was performed to assess for demographic trends. **Results:** The most common form of glaucoma treatment in Monroe County, NY was prescription drug therapy (36.82%), which was predominant across all racial, gender, and age groups. This was followed by laser surgery (3.26%), glaucoma drain (1.47%), and other forms of glaucoma surgery (0.58%). Women displayed a higher incidence of laser surgery, glaucoma drainage, and other glaucoma surgeries (3.58%, 1.77%, and 0.69%, respectively) with a lower incidence of prescription drug usage (36.14%) compared to men. Black non-Hispanic patients had a higher incidence of laser surgery and prescription drug usage (3.39% and 47.20%, respectively), but a lower incidence of glaucoma drainage and other glaucoma surgeries (1% and 0%, respectively) compared to other racial groups. **Conclusions:** This study elucidates the differences in glaucoma treatment types across different racial, gender, and age groups in Monroe County, NY. The results underscore the disparities in treatment rates for Medicare patients diagnosed with glaucoma in Monroe County. The results justify the need for increased interventions to increase access to a variety of glaucoma treatment options to mitigate disparities in glaucoma outcomes.

## 1. Introduction

Glaucoma, a condition that leads to irreversible blindness, is a major concern worldwide. It is caused by a group of optic neuropathies that cause chronic progressive degeneration of retinal ganglion cells that leads to peripheral and eventually central vision loss if left untreated. There are two main types of glaucoma: angle closure glaucoma (ACG) and open-angle glaucoma (OAG), based on the anatomy of the angle, with the most common form being primary open-angle glaucoma (POAG). The main mechanism leads to increased resistance to the flow of aqueous humor through the trabecular meshwork. This disease is quite insidious as patients are largely asymptomatic until they experience significant, permanent, peripheral, and eventual central visual loss if left untreated.

Glaucoma is associated with numerous risk factors, including older age, race and ethnicity, family history, decreased central corneal thickness, and elevated intraocular pressure. Additional risk factors include the presence of co-morbidities, such as diabetes and hypertension, as well as lifestyle factors such as tobacco smoking, inadequate exercise, and poor nutrition and many others [1]. Glaucoma in the United States affected nearly 2.2 million Americans in 2004, with the prevalence increasing to over 3 million in 2020. This increasing prevalence rate and disease progression resulted in vision impairment for over 120,000 individuals. With an aging population, this number is expected to increase to over 3.36 million persons affected by POAG by 2030. The Baltimore Eye Survey revealed that POAG is 6.6 to 6.8 times more prevalent among African Americans than Caucasians in some age groups [1,2,3,4].

Glaucoma is a major public health concern as it is one of the leading causes of irreversible blindness globally. Around the world, roughly 60 million individuals have glaucoma, and the prevalence is expected to surge to 110 million individuals by 2040 [2].

Glaucoma is evaluated by examinations in combination with diagnostic testing during routine eye examinations for an accurate assessment of disease severity. For example, data from tonometry, gonioscopy, and visual field testing, optical coherence tomography, and funduscopic examinations are all used in the diagnosis and management of glaucoma patients [5].

To date, there are no curative treatments for glaucoma. Instead, the management strategy involves early diagnosis and treatment to reduce the severity of vision loss. This is achieved by reducing the intraocular pressure to prevent further optic nerve damage. These treatments include topical eye drops and laser and incisional procedures [6].

This study seeks to specifically examine differences in glaucoma treatment rates that may be exacerbating disease severity and prognosis for individuals most acutely affected by glaucoma in Monroe County, NY, USA.

### 1.1. Disparities in Glaucoma Diagnosis

There are significant glaucoma racial health disparities that have been documented widely in the literature, as Black Americans are more likely to be diagnosed with glaucoma at a younger age, with a greater severity and a more rapid progression compared to White Americans [7]. For example, Black Americans are six times more likely to be become blind from POAG compared to White Americans [8,9]. Additionally, Acuff et al. found in the National Institute of Health-funded research program, All of Us, that Black Americans and other racial minorities are diagnosed with glaucoma, on average, 6 years earlier than White Americans [5]. Furthermore, the results from the African Descent and Glaucoma Evaluation Study (ADAGES) found that individuals with African descent had an increased risk of visual field loss and elevated intraocular pressure during follow-up [10].

Significant glaucoma racial health disparities also exist for the Latino population, who are individuals who are born into or who are descendants of a Spanish-speaking community. Latinos are the fastest growing and largest US minority group, who are expected to make up about a quarter of the US population by 2050 [11]. According to the Los Angeles Latino Eye Study (LALES), which is the largest population-based assessment of visual impairment, ocular disease, and visual functioning of Latinos to date, Latinos have a higher prevalence of glaucoma compared to non-Hispanic White Americans, and a similar prevalence when compared to Black Americans [12,13].

The research on glaucoma gender disparities is mixed. Overall, women tend to outlive and outnumber men. Additionally, women are the majority of the aging population. With respect to glaucoma prevalence, women share the greatest burden of glaucoma disease, as 60% of all glaucoma cases are women [2]. Multiple studies have demonstrated that women are at an increased risk of PACG due to anatomical differences. However, the literature is inconclusive regarding the gender risks associated with POAG. For example, both the Beaver Dam Eye Study and the Baltimore Eye Survey found no significant differences in the gender prevalence of POAG [3,4]. However, the Framingham Eye Study and the Rotterdam Study found an increased prevalence of POAG in males [14,15]. The Blue Mountains Eye Study found an increased prevalence of POAG in females [16]. Other studies have postulated a protective effect of prolonged endogenous estrogen exposure for glaucoma development. For example, studies have shown that women who underwent late menarche, early age of bilateral oophorectomy, who were multiparous, or underwent early menopause were associated with an increased risk of glaucoma. However, the gender differences of glaucoma risk are complex and may be explained by additional non-biological factors, including socioeconomics, culture, health beliefs, and access to high-quality health care [17,18].

### 1.2. Glaucoma Treatment

The timely intervention of glaucoma is imperative to prevent further progression to permanent vision loss. The cornerstone of glaucoma treatment includes decreasing the intraocular pressure. This can be achieved by various methods, including topical hypotensive eye drops, laser procedures, and incisional eye surgery. Surgical treatment options include trabeculectomy, drainage device implantation, and minimally or microinvasive glaucoma surgery [19].

Topical drug therapies are the mainstay of intraocular pressure reduction, which include many different medication classes such as prostaglandin analogues, beta-blockers, alpha adrenergic agonists, carbonic anhydrase inhibitors, cholinergic agents, nitric-oxides, and rho-kinase inhibitors [1]. Specifically, medications within the prostaglandin class were more effective at lowering the intraocular pressure for patients with POAG or ocular hypertension compared to drugs of other classes [20]. The Ocular Hypertension Treatment Study (OHTS) showed that the usage of topical hypotensive eye drops in individuals with elevated intraocular pressure can delay or prevent the onset of POAG [21]. Furthermore, the Early Manifest Glaucoma trial found that for every 1 mm Hg reduction in intraocular pressure, there is a 10% decreased risk of glaucoma progression [22]. These studies suggest that effective glaucoma management necessitates proper intraocular pressure control.

Laser procedures or incisional eye surgery are indicated when patients are refractory to or non-compliant with medical therapy. Laser trabeculoplasty is a common type of non-invasive, effective laser procedure that targets the trabecular meshwork to decrease the intraocular pressure [1]. The predominant types of trabeculoplasty are argon laser trabeculoplasty (ALT) and selective laser trabeculoplasty (SLT). ALT works by targeting an argon laser to the trabecular meshwork to induce mechanical and biological changes to decrease the outflow resistance of the aqueous humor. SLT works by targeting trabecular cells and reducing the intraocular pressure without inducing thermal or significant structural damage compared to ALT [23]. The most common type of incisional eye surgery is a trabeculectomy, also known as filtration surgery, which is the main surgical option to lower the intraocular pressure. This surgery involves excising part of the trabecular meshwork along with adjacent corneoscleral tissue to allow for the drainage of aqueous humor beneath the conjunctiva [1]. Tube shunts, also known as glaucoma drainage devices, are another type of incisional surgery. This involves placing an implant in the anterior, posterior or vitreous chamber to shunt aqueous humor away into a subconjunctival filtration bleb which overlies a scleral plate. There are various types of tube shunts, with the most common being the Molteno, Baerveldt, and Ahmed implants [23,24].

The latest glaucoma surgical treatment option is the minimally/microinvasive glaucoma surgery (MIGS). This treatment option works by increased aqueous humor outflow through the trabecular meshwork and Schlemm’s canal. Other mechanisms of decreasing the intraocular pressure include bypassing the trabecular meshwork via subconjunctival filtration. There are various types of MIGS procedures and devices, including the iStent inject, Hydrus, Trabectome, Kahook Dual Blade, and XEN Gel Stent. This surgical option is heralded as achieving greater surgical success due to decreased inflammation from micro incisions [1,25].

### 1.3. Disparities in Glaucoma Treatment

Overall, Black patients are undertreated for glaucoma, with a greater risk of suboptimal surgical outcomes. For example, Black Medicare beneficiaries had lower rates of glaucoma treatment compared to White Medicare beneficiaries. Even among Black Medicare patients who had an ophthalmology visit within the study year, they were still less likely to undergo medical or surgical treatment for glaucoma compared to White Medicare patients [26]. Furthermore, studies by Javitt et al. [8] and Devgan et al. [27] found that the observed rate of glaucoma surgeries for Black Medicare beneficiaries was lower than the expected rate of glaucoma surgery compared to White Medicare beneficiaries, given the overall higher prevalence of glaucoma in Black patients. Studies by Halawa et al. [28] found that Black Medicare beneficiaries had higher odds of glaucoma surgery compared to White beneficiaries, while Hispanic Medicare beneficiaries had higher odds of SLT compared to White beneficiaries. These findings suggest that Black patients may be undergoing glaucoma surgery at a delayed stage with a greater severity of disease, with a greater risk of surgical complications. Latino patients were found to have similar odds of glaucoma surgery as non-Hispanic patients, but with a greater severity of disease at the time of surgery [28]. When investigating the long term outcomes of several glaucoma surgeries, Black patients were found to be at greater risk for suboptimal surgical outcomes compared to White patients, including the need for reoperation, shunt failure, filtration failure, and elevated intraocular pressure during follow-up [29,30,31,32].

There have been multiple clinical trials to assess the efficacy of various glaucoma surgery options. For example, in the Advanced Glaucoma Intervention Study (AGIS), which included patients with refractory POAG aged 35 to 80 years old without previous glaucoma surgery, patients were randomized into one of two treatment sequences: ALT-trabeculectomy-trabeculectomy (ATT) or trabeculectomy-ALT-trabeculectomy (TAT), and evaluated for treatment failure. This study found that both Black and White participants were equally likely to achieve reduced intraocular pressure; however, Black participants had better long-term vision functional outcomes with the ATT sequence, while White participants had better long-term vision functional outcomes with the TAT sequence [33]. 

In the Tube Versus Trabeculectomy (TVT) Study, individuals aged 18 to 85 years old with uncontrolled glaucoma who had undergone previous cataract or glaucoma surgery were randomized to either the Baerveldt tube shunt or trabeculectomy with mitomycin-C (MMC) and assessed for surgical failure. At the 5-year follow-up time point, there were no statistically significant associations with regards to age, gender, race or ethnicity, and surgical failure risk [34]. In the Primary Tube Versus Trabeculectomy (PTVT) Study, individuals aged 18 to 85 with uncontrolled glaucoma who had not undergone previous intraocular surgery were randomized to either the Baerveldt tube shunt or trabeculectomy with MMC. Again, this study found no statistically significant associations regarding age, gender, race or ethnicity, and the risk of surgical failure at 5 years of follow-up [35]. 

### 1.4. Demographics of Monroe County, New York

Monroe County is located in the northern tier of western New York State. It is comprised of 19 towns, 10 villages, and the City of Rochester, which is New York’s 3rd largest city [36]. It was founded in 1821, has a population of 748,482 (2023), with a median household income of USD 71,450 (2022), and a poverty rate of 13.9% [36]. The racial demographics are the following: non-Hispanic White 68.9%, Black 14.8%, Hispanic or Latino 9.9%, Asian 3.8%, Multiracial 2.4%, and American Indian and Alaskan Native 0.2% [37]. The United States as a whole has a total population of 331,449,281 (2020), with a median household income of USD 74,755 (2022), and a poverty rate of 12.6% [38]. The racial demographics include: non-Hispanic White 58.9%, Hispanic or Latino 19.1%, Black 12.6%, Asian 6.1%, Multiracial 2.4%, American Indian/Alaskan Native 0.7%, and Native Hawaiian or Other Pacific Islander 0.2% [37]. 

Based on the Center for Disease Control (CDC) Vision and Eye Health Surveillance System data, in 2019, the crude prevalence of diagnosed glaucoma of any type in the Medicare data set in Monroe County was 11.71%, with higher rates in non-Hispanic Black, female, and 85 and older individuals [39,40]. Furthermore, according to the Health Resources and Services Administration’s Area Health Resources Files (AHRF), a national database on health care professionals, health facilities, hospital utilization, and hospital expenditures at the county, state, and national levels, there were a total of 83 active M.D. ophthalmologists in Monroe County, NY, USA, in 2023, with 10.99 M.D. ophthalmologists per 100,000 people in the county [41].

Based on the existing literature on glaucoma diagnosis and treatment disparities, we hypothesize that there are racial and gender disparities in treatment patterns for individuals with glaucoma in Monroe County, NY, USA.

### 1.5. Objective

The primary objective of this study is to analyze the glaucoma treatment data of Medicare beneficiaries’ claims from Monroe County, NY, using the National Vision and Eye Health Surveillance System (VEHSS) to determine whether differences in glaucoma treatment patterns exist within this patient population. In addition, we aim to compare New York State (NYS) and U.S. National trends within the VEHSS with Monroe County. Our secondary objectives include subgroup analyses by patient demographics, including age, gender, and race/ethnicity with respect to the glaucoma treatment type received. 

## 2. Methods

### 2.1. Data Source

We analyzed data from the Vision and Eye Health Surveillance System (VEHSS)—a program developed by Vision Health Initiative (VHI) of the Center for Disease Control (CDC) to assess the burden of ocular disease and the current ophthalmic care infrastructure in the United States [42]. The goal of the VEHSS is to inform eye care professionals, policy makers, and researchers on existing disparities within vision care. The data include retrospective data analyses from multiple sources to generate composite incidence rates of various diseases and treatments. These analyses include de-identified information including diagnoses, treatments, and demographic data. We utilized data consisting of Medicare enrollees diagnosed with glaucoma who had submitted treatment claims. 

### 2.2. Study Population

Medicare enrollees with glaucoma in Monroe County who received glaucoma prescription drugs, laser surgery, glaucoma drainage implant, and glaucoma surgery (goniotomy, trabeculectomy, iridectomy, scleral reinforcement, or ciliary body destruction) in 2019 were included. Data were stratified by age, gender, and racial/ethnic groups including Asian, non-Hispanic Black/African American, any-race Hispanic, and non-Hispanic White. Only data from 2019 were included as county-level data for years prior were not available. Census data for Monroe County are as described above. The primary outcome of measure was the incidence rate for a specific treatment defined as the percentage of patients within a given demographic group diagnosed with glaucoma who had that treatment claim.

The main aim of this study is to determine the distribution of glaucoma treatment types in Monroe County, NY, USA. We conducted an analysis to identify potential risk factors such as race/ethnicity, age, and gender. Additionally, descriptive data analysis was carried out to identify any demographic trends.

### 2.3. Statistical Analyses

We conducted sensitivity analysis to compare treatment types in Monroe County in 2019 across different ages, racial groups, and genders using Fisher’s exact test. In all cases, *p*-values of ≤0.001 were considered statistically significant. In some cases, missing data made it difficult to directly compare treatment choices. In those cases, we assumed a wide range for the missing data (from 0 to 10).

## 3. Results

**Figure 1:**
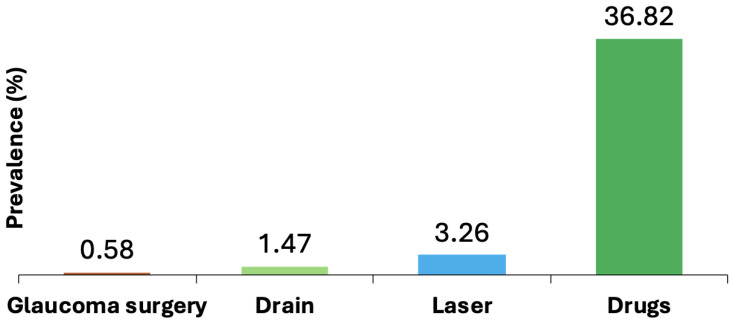
Comparing all Glaucoma treatments in Monroe County (2019).

The most common form of glaucoma treatment in Monroe County, NY, was prescription drug therapy (36.82%), which was predominant across all racial, gender, and age groups. This was followed by laser surgery (3.26%), a glaucoma drain (1.47%), and other forms of glaucoma surgery (0.58%). These results are summarized in Figure 1.

**Figure 2:**
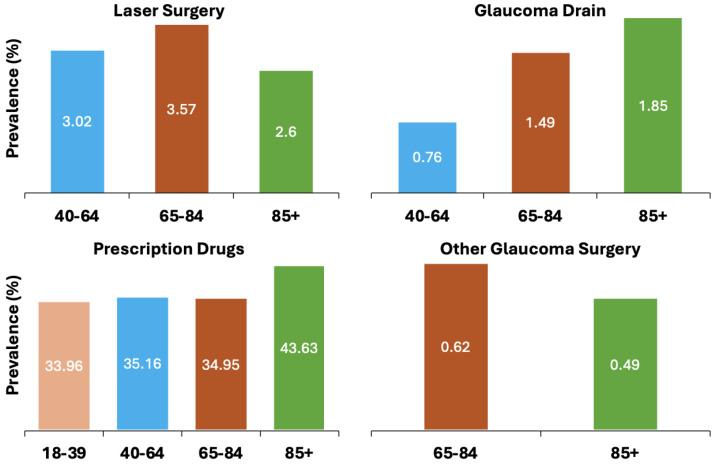
Trends across age groups for Glaucoma treatment in Monroe County (2019).

Individuals aged 85 and above exhibited a higher incidence of glaucoma drainage and prescription drug usage (1.85% and 43.63%, respectively) compared to individuals aged 18 to 84. Individuals aged 65 to 84 had the highest rates of laser surgery and other forms of glaucoma surgery (3.57% and 0.62%, respectively) than other age groups. Figure 2 and Table 1 shows these trends across age groups for various treatments.

**Figure 3:**
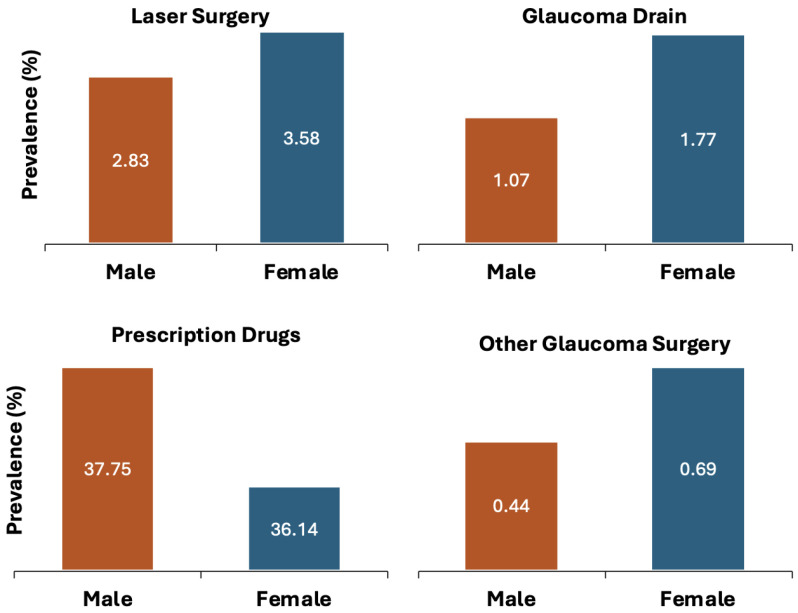
Trends across genders for Glaucoma treatment in Monroe County (2019).

In general, women displayed a higher incidence of laser surgery, glaucoma drainage, and other glaucoma surgeries (3.58%, 1.77%, 0.69%, respectively) with a lower incidence of prescription drug usage (36.14%) compared to men. In other words, females had higher incidences for all types of glaucoma treatments except for prescription drug usage compared to men, as seen in Figure 3 and Table 2.

**Figure 4:**
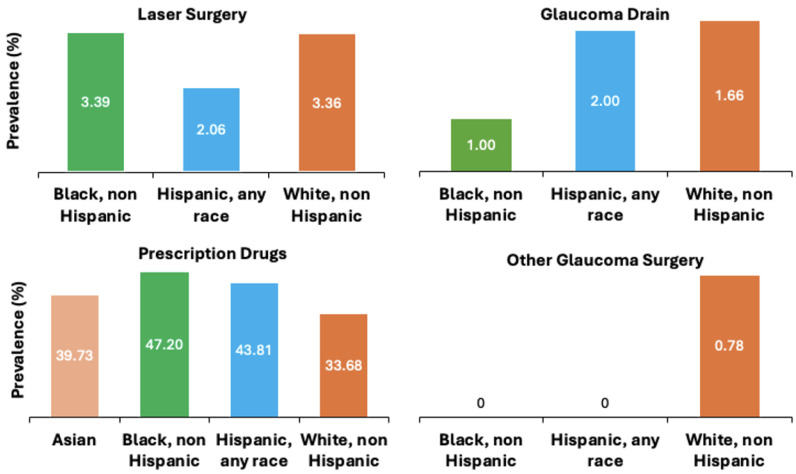
Trends across racial/ethnic groups for Glaucoma treatment in Monroe County (2019).

Trends across racial/ethnic groups showed that Black non-Hispanic patients had a higher incidence of laser surgery and prescription drug usage (3.39% and 47.20%, respectively), but a lower incidence of glaucoma drainage and other glaucoma surgeries (1% and 0%, respectively) compared to other racial groups. In fact, only non-Hispanic White patients were found to have undergone other forms of glaucoma surgery (0.78%). In addition, non-Hispanic Whites had the lowest rates of prescription drugs as treatment for glaucoma (33.68%). Racial/ethnic trends for glaucoma treatment are shown in Figure 4 and Table 3.

**Figure 5:**
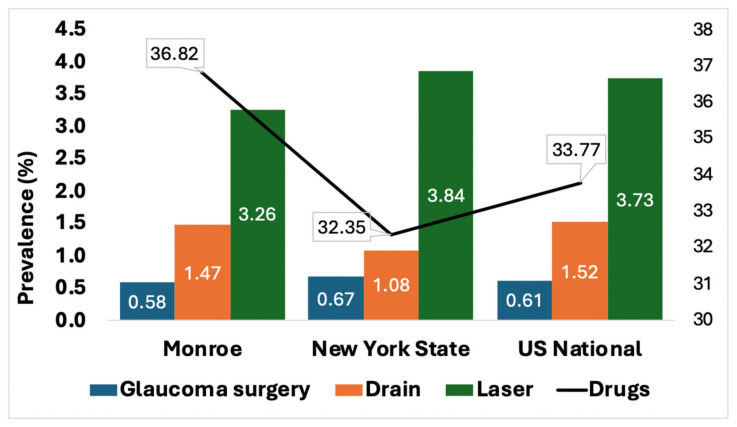
Comparison of Glaucoma treatments in Monroe County vs. New York State vs. US National.

Figure 5 shows the incidence of glaucoma treatments for Monroe County in comparison to NYS and U.S. National. Monroe county had lower rates of laser (3.26%) and other glaucoma surgery (0.58%) than both NYS (3.84% and 0.67%, respectively) and U.S. National (3.73% and 0.61%). At the same time, Monroe County had higher rates of glaucoma drug prescription (36.82%) than both NYS (32.35%) and U.S. National (33.77%). NYS had the lowest rates of glaucoma drain (1.08%) among these three groups.

Sensitivity analyses were conducted and are shown in Table 1, Table 2 and Table 3 for trends in age, gender, and race, respectively. There were significant differences in the treatment type between age and racial groups in Monroe County in 2019 (*p* < 0.001). However, differences between genders were not significant (*p* = 0.2009).

## 4. Discussion

This study elucidates the differences in glaucoma medical and surgical treatment patterns in Monroe County, New York, with respect to race and gender. As a microcosm of the United States, Monroe County represents the issues facing glaucoma treatment for marginalized communities. A multi-pronged approach that addresses key barriers to eye care, and more specifically, glaucoma care, appears to be the most logical path forward. By doing so, we will be able to combat the formidable challenges that have created the racial and gender disparities in glaucoma treatment. Some of these evidence-based interventions include adopting teleophthalmology services, mobile eye clinics, and culturally relevant community outreach programming in order to reach individuals who may not historically have access to high-quality ophthalmology departments [43]. For example, as transportation is a documented barrier to glaucoma follow-up care, giving patients the option to attend virtual appointments via teleophthalmology or bring glaucoma care directly to patients in need via mobile clinics can serve to reduce the logistical or financial burden related to transportation. Additionally, greater investments in Black and Brown communities through culturally specific programming can aid in creating strong community engagement. By doing so, this will help individuals become more knowledgeable about their eye diseases so that they will be more likely to seek treatment if needed [44].

Multiple factors may explain the racial disparities in glaucoma treatment patterns in Monroe County, NY. For example, according to the CDC Vision Health Initiative, a low income, low educational attainment, food insecurity, and neighborhood safety may all be significant social determinants of health that can contribute to adverse vision and eye health outcomes. Furthermore, multiple studies have shown the relationship between a low socioeconomic status with visual impairment, blindness, and sudden vision loss. Additionally, studies have also found associations between a low socioeconomic status and the lower utilization of eye and vision care [13,45].

Furthermore, researchers have studied specific barriers related to glaucoma care. For example, in a randomized control trial in Philadelphia, Black patients with glaucoma reported forgetfulness, a lack of adequate transportation, and an inability to miss work as reasons for missing follow-up ophthalmology appointments [46]. Other studies of Black patients with glaucoma in New Haven cited the following as reasons for a lack of glaucoma follow-up care: limited access to a car, current smoker status, living alone, and prolonged periods between screening and a comprehensive evaluation [47,48].

Additionally, medical treatment non-adherence has been documented in the literature as a significant barrier to successful glaucoma management. The Glaucoma Adherence and Persistency Study found that more than 90% of patients with glaucoma did not regularly refill their medical prescriptions during their first year of treatment [49]. This is especially true for Black and Latino patients, as they are more likely to have poor medical adherence and are less likely to attend follow-up visits with their providers compared to White patients [50]. A few studies have quantified this difference, where White patients are three times more likely than Black patients to be more than 80% compliant with their medication regime [51,52,53]. There are many factors that may explain differences in medication adherence including a lack of effective communication between the physician and patient. For example, studies suggest that communication between physicians and patients of different races is less positive and productive compared to communication between physicians and patients of the same race [54,55,56]. Therefore, enhanced education and awareness around this issue is critical to improving communication and medical adherence in Black and Latino populations. Furthermore, a diverse ophthalmology workforce with increased physician–patient racial concordance may be able to achieve similar goals.

This study has a few limitations. As we primarily relied on VEHSS Medicare data, this restricted our analysis to only a subset of the entire data available. Furthermore, this study focused primarily on data for Monroe County; we did not analyze data for other counties in western New York and only aggregate data were used for NYS. Future studies include expanding our geographical analysis to other counties in western New York and New York State to obtain a more comprehensive understanding of treatment patterns with respect to race and gender. Furthermore, the VEHSS does not analyze trends across socioeconomic factors such as the income level or education level, and geographical stratification is limited to the county level. Utilizing a data source that includes these factors and stratifies according to Zip code, for example, may help uncover further disparities within glaucoma treatment.

## 5. Conclusions

In conclusion, this study highlights the differences in glaucoma treatment types across different racial, gender, and age groups in Monroe County, New York. The results underscore the disparities in treatment rates for Medicare patients diagnosed with glaucoma in Monroe County. The results justify the need for increased interventions to increase access to a variety of glaucoma treatment options to mitigate these disparities in glaucoma treatment. In addition to education and awareness, addressing social determinants of health and diversifying the ophthalmologic workforce as well as working with other physicians, para-professionals, and stakeholders will be crucial to improving access and adherence to glaucoma treatment.

## Figures and Tables

**Table 1:** Glaucoma treatment by age in Monroe County (2019).

AGE	TREATMENT				NO TREATMENT	TOTAL	
	Drain	Laser	Drugs	Other			
**40–64**	4	16	186	0	324	530	*p* < 0.0001 (Fisher’s exact test)
**65–84**	35	85	838	14	1428	2400	
**85+**	14	21	353	3	419	810	

**Table 2:** Glaucoma treatment between gender groups in Monroe County (2019).

GENDER	TREATMENT				NO TREATMENT	TOTAL	
	Drain	Laser	Drugs	Other			
**Male**	17	45	604	7	927	1600	*p* = 0.2009 (Pearson’s chi-square test)
**Female**	38	79	795	15	1274	2200	

**Table 3:** Glaucoma treatment across race groups in Monroe County (2019).

RACE	TREATMENT				NO TREATMENT	TOTAL	
	Drain	Laser	Drugs	Other			
**Black, non Hispanic**	4	23	320	0	333	680	*p* <0.0001 (Fisher’s exact test)
**Hispanic, any race**	2	3	83	0	102	190	
**White, non Hispanic**	46	94	943	21	1696	2800	

## Data Availability

The data is available on the CDC website free VEHSS data source.
